# Valproic acid for treatment of traumatic brain injury: Study protocol for the VIBRANT prospective randomized trial

**DOI:** 10.1111/trf.70029

**Published:** 2025-12-20

**Authors:** Maxime A. Visa, Marjorie R. Liggett, Sharnia Lashley, Umar Bhatti, Zaiba A. Dawood, Alvin Anand, Nathan P. Gill, Denise M. Scholtens, Bowen Wang, Hasan B. Alam

**Affiliations:** ^1^ Department of Surgery Northwestern University Feinberg School of Medicine Chicago Illinois USA; ^2^ Department of Surgery, Cedars Sinai California Los Angeles USA; ^3^ Department of Preventive Medicine, Feinberg School of Medicine Northwestern University Chicago Illinois USA

**Keywords:** brain, clinical trial outcomes, neuroprotective, trauma, valproic acid

## Abstract

**Background:**

Traumatic brain injury (TBI) carries significant mortality and morbidity in civilian and military populations. Current treatment guidelines for TBI are primarily supportive, and no pharmacological agent exists to attenuate the progression of brain injury. Valproic Acid (VPA) has long been used to treat neurological disorders; however, recent work has demonstrated its potential as a neuroprotective agent. We have already demonstrated that VPA administration in swine models of TBI (with or without associated hemorrhage and polytrauma) significantly improves survival and neurological recovery and decreases brain lesion size compared to controls. This paper introduces a phase 2/3 clinical trial that is designed to evaluate the efficacy and safety of VPA administration in patients with TBI.

**Methods:**

In this randomized, double‐blind, placebo‐controlled, multicenter trial, patients with moderate to severe TBI (GCS 3–12) across nine level 1 trauma centers in the US will be randomized to receive either standard of care treatment and 250 mL of isotonic saline (control), or standard of care treatment and intravenous VPA at either 50 mg/kg (low‐dose VPA group), or 100 mg/kg (high‐dose VPA group). The primary endpoint of this clinical trial will be neurological status as measured by the Extended Glasgow Outcome Scale (GOS‐E) 3 months post‐TBI.

**Discussion:**

Our team has conducted multiple large animal studies that strongly support the cytoprotective effects of VPA treatment. The goal of this upcoming trial is to study the efficacy and safety of two doses of VPA in patients with moderate to severe TBI.

**Trial registration:**

ClinicalTrials.gov, https://clinicaltrials.gov/study/NCT07166393, September 3, 2025.

**Abbreviations**
AE: adverse effects
aPTT: activated partial thromboplastin time
BMI: body mass index
c‐IRB: common institutional review board
CDMRP: congressionally directed medical research program
CT: computed tomography
DCR: damage control resuscitation
DLT: dose‐limiting toxicities
DMC: data monitoring committee
DRS: disability rating score
DSMB: data safety monitoring board
EFIC: exception from informed consent
FDA: Food and Drug Administration
GABA: gamma‐aminobutyric acid
GAD: glutamic acid decarboxylase
GCS: Glasgow coma score
GOS‐E: extended Glasgow outcome score
HAT: histone acetyltransferases
HDAC: histone deacetylases
HDACi: histone deacetylase inhibitor
HPC: hemorrhagic progression of the contusion
ICAM: intracellular adhesion molecule
ICP: intracranial pressure
INR: international normalized ratio
IT: information technology
ITT: intent‐to‐treat
IV: intravenous
K_2_EDTA: dipotassium ethylenediaminetetraacetic acid
LAR: legally authorized representatives
MICE: multiple imputation by chained equations
NS: normal saline
NSS: neurological severity score
NUCATS: Northwestern University Clinical and Translational Sciences Institute
NUDACC: Northwestern University data analysis and coordinating center
NURIPS: Northwestern University research image processing system
PBMC: peripheral blood mononuclear cell
PD: pharmacodynamic
PFC: prolonged field care
PI: principal investigator
PK: pharmacokinetic
POD: post‐operative day
PT: prothrombin time
s‐IRB: single institutional review board
SRC: safety review committee
TBI: traumatic brain injury
VIBRANT: valproic acid for traumatic brain injury trial
VPA: valproic acid

## 1 INTRODUCTION

Traumatic brain injury (TBI) is a major contributor to trauma‐related death and carries a significant long‐term morbidity burden. Nearly 2 million Americans suffer from new TBIs every year, and 50 million people worldwide are estimated to suffer from TBI annually, resulting in a global economic burden of $400 billion.^1^, ^2^ While this is important among civilian trauma patients, the prevalence and significance of this injury are particularly impactful among military service members. It has been established that TBI is a leading cause of battlefield mortality.^3^ Additionally, non‐fatal TBI is common in veterans, with more than 400,000 U.S. service members having suffered a TBI between the years of 2000 and 2022.^4^

Hemorrhage and TBI are the leading causes of death in the combat setting, where 87% of deaths occur before reaching a medical facility.^5^ Often, hemorrhagic shock and TBI are present together and in conjunction with other injurious mechanisms. The difficulty of treating TBI is underscored in these poly‐trauma patients, where different afflictions necessitate discrepant treatment goals. While TBI management guidelines recommend maintaining an elevated blood pressure to mitigate brain injury secondary to the primary insult, ongoing hemorrhage requires keeping blood pressure low.^6^, ^7^ While this scenario is challenging even in well‐equipped settings, the complexity of this situation is amplified in resource‐limited environments.

Current management guidelines for TBI are largely supportive, with no treatment options that specifically target the injured brain and improve neurologic and functional outcomes.^8^ Management of TBI patients—particularly those that present with other traumatic injuries—is resource‐intensive and requires team‐based, round‐the‐clock monitoring that is limited in austere military environments. While hospitals are typically well‐equipped to manage critically ill patients, military medicine can be marked by fewer personnel, equipment, and therapeutic options. The complexity and difficulty of treating TBI patients in military settings are expected to increase, with Department of Defense (DOD) predictions that future near‐peer conflicts will be marked by contested airspace hindering extrication to military treatment facilities.^9^ This change is anticipated to increase the time spent in prolonged field care (PFC) compared to past conflicts. Thus, there is a clear need to not only identify a safe and effective neuroprotective agent capable of mitigating underlying injurious processes of TBI, but one that can also be administered in austere environments. Such an agent could allow civilian and military providers alike to treat TBI while providing significant logistical benefits in the setting of PFC.

Over the past decade, our team has extensively investigated the role of valproic acid (VPA), a Food and Drug Administration (FDA)‐approved small molecule drug, as a potential treatment for TBI across numerous preclinical models. Between our translational models and confirmatory dose‐escalation models in humans, we have collected sufficient data on preclinical efficacy and clinical safety to justify a clinical trial aimed at evaluating VPA treatment in TBI patients. In this paper, we will discuss the proposed mechanism of VPA as a neuroprotective agent, discuss the relevant preclinical and clinical data supporting VPA as a potential treatment for TBI, and provide details of our upcoming randomized, placebo‐controlled, multi‐institutional clinical trial.

## 2 RATIONALE FOR VALPROIC ACID TREATMENT

**FIGURE 1:**
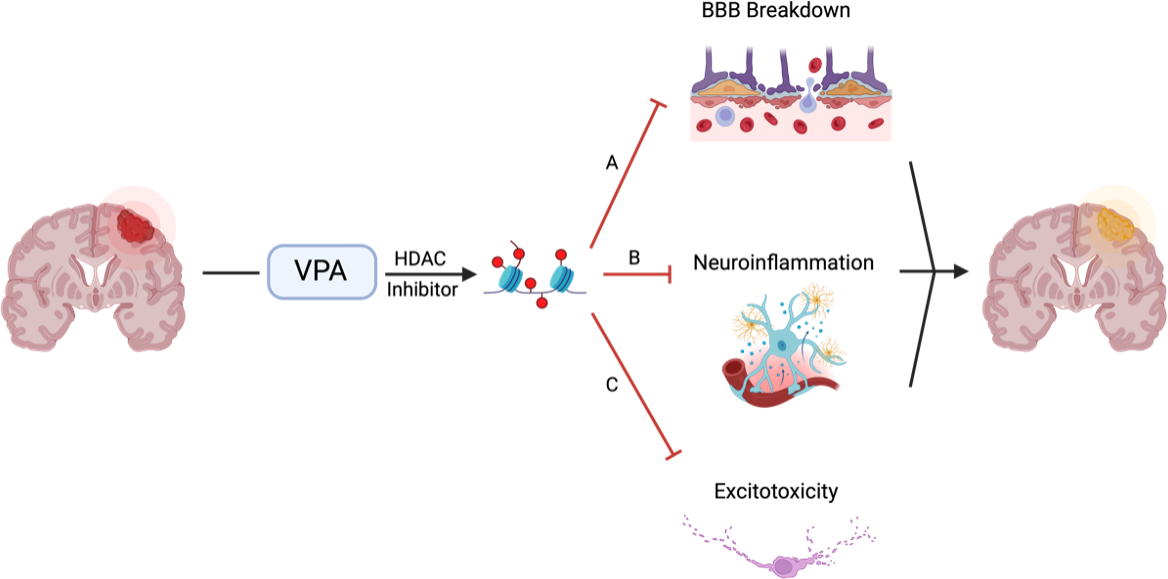
Proposed mechanism of traumatic brain injury (TBI) attenuation by valproic acid (VPA) based on murine and swine studies. As a histone deacetylase inhibitor (HDACi), VPA confers numerous neuroprotective effects by preventing the deacetylation of key nuclear and cytoplasmic proteins involved in cell survival and anti‐inflammatory pathways. (A) VPA has been shown to help maintain the integrity of the blood–brain barrier (BBB) following TBI, protecting neural parenchyma and curtailing edema. (B) VPA downregulates pro‐inflammatory mediators and cytokines, mitigating neuroinflammation. (C) VPA reduces presynaptic glutamate release and enhances GABAergic transmission to diminish excitotoxicity.

VPA has long been established as a treatment for a multitude of neurological and psychiatric disorders. Since its approval by the FDA in 1978, VPA is primarily used as a standalone or adjunctive therapy in treating various seizure types in both adult and pediatric patients, and has been recently repurposed for conditions such as bipolar disorder, diabetic peripheral neuropathy, postherpetic neuralgia, and migraine prophylaxis.^10^ Although its mode of action is multifactorial, recent studies have established VPA as a direct antagonist of histone deacetylases (HDAC), a class of epigenetic “erasers” that modulate gene expression through histone acetylation when given in higher doses. We have demonstrated that VPA is a non‐selective histone deacetylase inhibitor (HDACi) that can rapidly and reversibly increase the acetylation of nuclear and cytoplasmic proteins.^11^ Given the profound role of the histone epigenetic machinery in TBI pathogenesis and progression, the process of acetylation provides an attractive therapeutic target for the attenuation of brain injury because it is rapid, reversible, and modifiable^12^ (Figure 1).

In numerous studies across several animal models, we have shown that a single dose of VPA can improve survival and mitigate organ damage in models of lethal hemorrhage,^13^ poly‐trauma,^14^, ^15^ septic shock,^16^ ischemia‐reperfusion,^17^ and TBI.^18^ We first tested the protective role of VPA (300 mg/kg) following hemorrhagic shock in rat models subjected to 60% blood‐volume loss. There, we found that VPA boasted a survival rate of 75% compared to 25% in the control group.^13^

When we tested the neuroprotective effects of VPA alone for TBI in swine models, we found that a single dose of VPA (150 mg/kg) significantly improved survival in lethal TBI injury (83%) compared to animals that were resuscitated with normal saline (17%).^15^ Additionally, post‐TBI VPA treatment significantly ameliorated brain lesions, neuroinflammation, and edema while circulating levels of TBI biomarkers (e.g., GFAP) were also diminished.^18^, ^19^ Given that TBI and hemorrhagic shock frequently present simultaneously, we conducted experiments to determine the neuroprotective effect of VPA in combined TBI and hemorrhagic shock in a swine model. In this set of experiments, swine were subjected to controlled TBI and 40% estimated total blood volume loss. In this combined TBI and hemorrhagic shock model, animals that were treated with standard resuscitation augmented with VPA demonstrated significantly quicker neurological recovery, increased cognitive function, and smaller brain lesion sizes at POD 3 and POD 10 compared to the standard resuscitation group.^20^ In another experiment testing the effect of VPA on hemorrhagic shock, we demonstrated that animals subjected to lethal hemorrhage (50% estimated blood volume) had significantly better outcomes when treated with VPA and standard damage control resuscitation (DCR) protocol (80% survival) versus DCR alone (20% survival).^21^

We also tested VPA in a model of simulated PFC where blood transfusion was delayed for 72 h, and showed that animals treated with VPA required significantly less crystalloid resuscitation, had fewer post‐operative neurological impairments, and had a smaller brain lesion size compared to controls.^21^, ^22^

In addition to immediate neuroprotection, VPA has also been shown to enhance long‐term healing of the injured brain as seen by decreased neural apoptosis, inflammation, and degenerative changes, and improved neural plasticity 30 days post‐TBI.^23^ Pharmacokinetic (PK) data from our dose‐escalation trial show that the concentration of free VPA at the end of infusion strongly correlates with Neurological Severity Score (NSS), with doses as low as 50 mg/kg conferring neuroprotective effects.^24^ We anticipate that PK data obtained during our upcoming clinical trial can be used to further fine‐tune the optimal dosing regimen for use in trauma patients.^24^ In summary, across our numerous experiments studying the neuroprotective effects of VPA in animal models, it has become increasingly evident that VPA has the potential to be an effective and safe therapy for TBI in trauma patients.

## 3 DOSE CONSIDERATIONS

**FIGURE 2:**
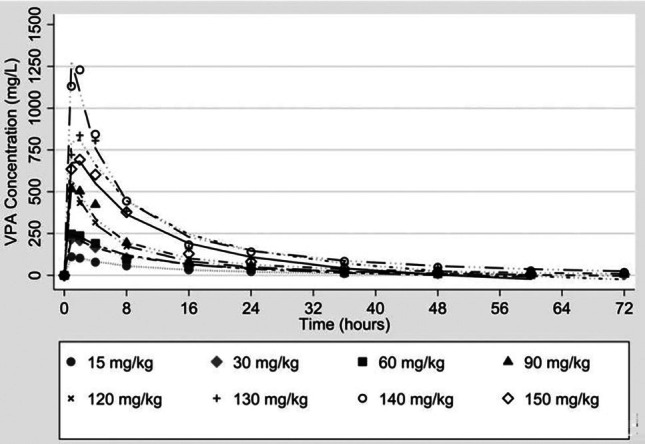
Plasma VPA levels over time in different dose cohorts (*n* = 6 per cohort, except the 150 mg/kg group, which had two subjects).^26^

Typical VPA dosing as an anti‐seizure agent ranges between 15 and 60 mg/kg/day. However, animal studies show that much higher doses (100–300 mg/kg) are needed in the setting of lethal hemorrhage to improve survival. To determine the lowest VPA dose that would offer a survival benefit following lethal injury, we completed a dose‐escalation study in a model of lethal polytrauma, hemorrhagic shock, and traumatic brain injury in swine.^15^ There, we discovered that VPA administered as a single IV dose of 150 mg/kg over 3 h significantly improved survival (83% vs. 17% in control; *p* < .05) and induced protective changes in the proteome that mediate cellular ischemia and inflammation. Follow‐up studies in non‐lethal injury models, however, suggest that a lower dose of 50 mg/kg may confer a similar level of neuroprotection as 150 mg/kg.^24^ To ensure the safety of higher doses of VPA in humans, we performed a double‐blind, placebo‐controlled Phase I clinical trial,^25^ where escalating doses of VPA were administered over 60 min via intravenous infusion in 59 healthy human subjects starting at 15 mg/kg and going up to 140 mg/kg (Figure 2). Subjects reported mild adverse effects (AEs)—including hypoacusis, chills, headache, tinnitus, and nausea—which were not different between the three highest dose cohorts (120, 130, and 140 mg/kg), and resolved within 12 h of infusion. Given that our proposed doses for this clinical trial will be less than these AE‐inducing doses (i.e., 50 and 100 mg/kg as described below), these findings suggest that the potentially beneficial effects of VPA significantly outweigh the mild side effects in the setting of severe injuries. Further, since our clinical trial requires only a single dose of VPA, dose‐limiting toxicities (DLTs) associated with long‐term VPA administration (or multiple doses) are not relevant.^27^, ^28^ Like most drugs, the circulating levels of VPA are also affected by body mass and surface area, and animal data must be converted into human dose equivalents.^29^ When we tested the serum levels of VPA in humans who had received doses ranging between 60 and 120 mg/kg, we found them comparable to levels in swine that were given doses as high as 300–400 mg/kg. Analyzing the human and swine dosing data together suggests that a swine dose of 100–150 mg/kg may be equivalent to a calculated human dose of 60–90 mg/kg. Therefore, we are proposing to test two doses of VPA: a lower (50 mg/kg) and a higher dose (100 mg/kg) of VPA against the standard of care.

## 4 VALPROIC ACID FOR TRAUMATIC BRAIN INJURY TRIAL (VIBRANT) CLINICAL TRIAL

The objective of this trial is to determine the efficacy of the administration of two different VPA doses in the acute setting in patients who have experienced moderate to severe TBI. We hypothesize that early administration of VPA will result in better neurological outcomes and long‐term recovery. While the exact mechanism behind VPA's neuroprotective effects in TBI patients remains unknown, there is adequate evidence supporting VPA's cytoprotective effects in the acute trauma setting to justify a phase 2/3 clinical trial. We received FDA approval (IND# 146723) for a randomized, double‐blinded, placebo‐controlled, multicenter superiority trial to evaluate the efficacy, safety, tolerability, pharmacokinetics, and pharmacodynamics of VPA in subjects with moderate to severe traumatic brain injury due to blunt trauma, which we have short‐named the **V**alproic Ac**I**d for Traumatic **BRA**in I**N**jury **T**rial (VIBRANT). This phase 2/3 clinical trial will be conducted across nine academic level 1 trauma centers across the United States, and is designed to evaluate the safety and efficacy of early administration of VPA in patients with moderate (Glasgow coma scale [GCS] 9–12) to severe (GCS 3–8) TBI relative to the current standard of care.

Enrolled patients with moderate to severe TBI as rated on the Glasgow Coma Scale between 3 and 12 due to blunt trauma will be randomized to the control arm, where they will receive standard‐of‐care treatment as defined in the Brain Trauma Foundation Guidelines in addition to a normal saline (250 mL 0.9% sodium chloride solution) vehicle, or the experimental arm where they will receive standard of care treatment and a single intravenous administration of VPA (Depacon®) at a dose of either 50 mg/kg (low‐dose VPA group) or 100 mg/kg (high‐dose VPA group) infused over 1 h.^8^ Treatment will be started within 120 min of the onset of the injury, and infusion will begin as soon as TBI is confirmed on the CT scan. All supportive care, imaging studies, and surgical interventions will adhere to the best clinical care guidelines.^8^, ^30^ Patients enrolled in the control arm will receive a 250 mL 0.9% sodium chloride solution injection that will be prepared similarly to, and visually resemble the study drug. Additionally, the saline control will be administered identically to VPA in the experimental arm. The purpose of including placebo‐treated subjects is to ascertain whether any abnormalities observed are due to VPA or environmental conditions and study procedures.

Given that this trial's intervention—a single dose of VPA (50 or 100 mg/kg) administered over 1 h—is completed once at the time of initial treatment, discontinuation of intervention does not apply to our trial. Following the single administration of VPA, there will be no ongoing or repeated interventions unique to the experimental arms; all subsequent interventions will follow standard TBI and resuscitation management protocols and will be provided equally to all enrolled patients at the discretion of the attending physician. If four patients experience adverse or serious events following VPA administration that are probably or definitely related to the study intervention, a potential trial enrollment pause will take place to perform a safety review. Investigators must use caution when administering concomitant therapies (Table S1), as VPA exhibits well‐known and defined drug interactions with several classes of therapeutics. Notably, due to hepatic clearance of VPA, drug–drug interactions may likely become less important over the course of hospitalization and should be considered by participating physicians and pharmacists. The administration of VPA is independent of posttraumatic seizure prophylaxis or treatment, and other adjuncts should be used for this purpose if desired or necessary. Past studies exploring VPA's use in the context of posttraumatic seizure mitigation were inferior compared to phenytoin.^31^ This trial does not have any provisions for ancillary or post‐trial care to enrolled patients.

Males or females between the ages of 18 and 65 years with mild to severe TBI (GCS 3–12) due to blunt trauma will be enrolled in this trial. To minimize heterogeneity, we will limit enrollment to patients with the initial CT scan showing at least one of the findings associated with Brain Injury Guideline (BIG 3) criteria, such as multiple locations of intraparenchymal hemorrhage (or one location ≥8 mm), scattered subarachnoid hemorrhage, displaced skull fracture, intraventricular hemorrhage, and sub dural or epidural hematoma ≥8 mm.^32^ Body Mass Index (BMI) must be between 18 and 35 kg/m^2^, and females must be surgically sterilized, postmenopausal, or have a negative urine pregnancy test. Only individuals who meet all the aforementioned criteria will be enrolled in this trial. Patients with a known history of adverse reactions to VPA, a known history of Hepatitis B or C, or a clinical history of hepatic dysfunction, pancreatitis, or renal insufficiency will be excluded from this trial. Further, we will exclude patients with a known history of thrombocytopenia, a platelet count less than 100,000 per microliter of blood, 2nd or 3rd‐degree burns of any size and location, patients whose time of injury is unknown, patients who have non‐survivable injuries in the estimation of the attending trauma surgeon, or those who have a known “do not resuscitate” order before randomization. Additionally, patients who are currently incarcerated or in police custody and interfacility transfers are excluded from eligibility. Pregnant or lactating patients, those with inadequate venous access, and patients who are in hemorrhagic shock with a systolic blood pressure of <90 mmHg on initial evaluation in the emergency department are excluded. If treatment cannot start within 120 min from the onset of the injury, and there is greater than 90 min between the onset of injury and arrival at the hospital, the patient will be excluded. If the patient is enrolled in another clinical trial or if the patient has opted out of the trial through a research “opt‐out” bracelet, the patient will also be excluded.

**FIGURE 3:**
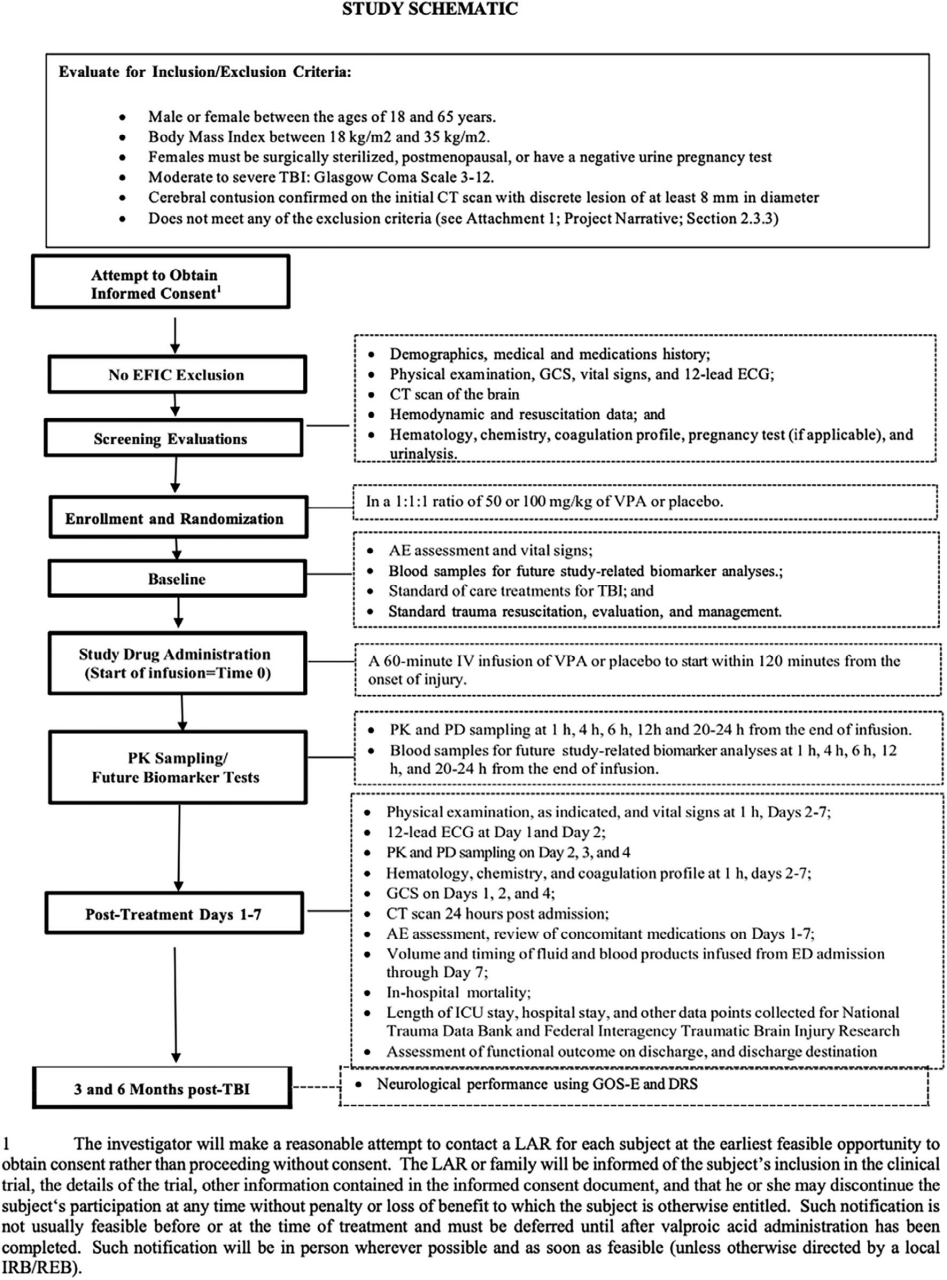
Study schematic for the VIBRANT trial.

The primary endpoint in this study will be the GOS‐E at 3 months post‐injury. Secondary endpoints include hemorrhagic progression of the contusion (HPC) (as measured by follow‐up CT scan over the first 24 h post‐injury), and Disability Rating Score (DRS) at discharge and at 3 months post‐injury. Exploratory endpoints will include 6‐month DRS and GOS‐E, as well as GCS 24 h post‐injury. In addition, we will monitor PK and pharmacodynamic (PD) data of VPA in the injured patient population as well as coagulation parameters including Prothrombin Time (PT), Activated Partial Thromboplastin Time (aPTT), International Normalized Ratio (INR), as well as Thromboelastography (whenever available). Endothelial markers including serum syndecan‐1, E‐selectin, and Intercellular adhesion molecule [ICAM]‐1 will be measured as well. Sub‐stratification of TBI severity into moderate (GCS 9–12) and severe (GCS 3–8) categories will also be assessed as exploratory endpoints (Table S2). The timing of patient enrollment is depicted in Figure 3.

## 5 STATISTICAL METHODS

Power analyses indicate that up to 432 patients randomized to three equally sized treatment groups is sufficient to achieve a power of at least 80% under a variety of treatment effect scenarios with 5% Type I error under two‐sided testing, accounting for an attrition rate of 5% (Tables S3 and S4). Patients will be allocated to the experimental or either treatment arm in a 1:1:1 fashion using a centralized, online, randomized system across all participating sites. Randomization will be stratified according to TBI severity and clinical site. Random block sizes of 3, 6, and 9 will be used.

The primary endpoint, 3‐month GOS‐E, will be analyzed using an ordinal logistic regression model, where GOS‐E will be the response variable. The treatment group will be the primary predictor of interest, with site and TBI severity included as covariates since they are stratification factors for randomization. To determine if GOS‐E differs between any pair of the three treatment groups, a second model will be fit without the treatment group as a predictor and compared to the first model using a likelihood ratio test. If the test is significant at α = 0.05, each pair of treatment contrasts (3 total: control vs. 50 mg, control vs. 100 mg, 50 vs. 100 mg) will be tested separately for GOS‐E differences. The ordinal logistic regression models will be fit using the polar function in the MASS R package. This analysis will be performed using an intent‐to‐treat (ITT) principle, in which the treatment group variable corresponds to the arm to which an individual was randomized, regardless of protocol adherence. Additionally, as a sensitivity analysis, we will perform an as‐treated analysis, in which the treatment group variable corresponds to the treatment an individual actually received.

The secondary outcomes can all be treated as continuous variables and will therefore be analyzed using linear regression models. The structure of the analysis will be similar to that of the primary outcome. Specifically, for each secondary endpoint, a linear regression model will be fit with that endpoint as the response variable, with treatment group as the primary predictor of interest, with site and TBI severity as additional covariates. To determine if this endpoint differs between any pair of the three treatment groups, a second model will be fit without treatment group as a predictor and compared to the first model using a likelihood ratio test. If the test is significant at α = 0.05, each pair of treatment contrasts (3 total: control vs. 50 mg, control vs. 100 mg, 50 vs. 100 mg) will be tested separately for differences in the given secondary endpoint. The linear regression models will be fit using the lm function in R. As with the primary endpoint, the secondary endpoints will be analyzed according to the ITT principle, with an as‐treated sensitivity analysis.

Exploratory endpoints will be analyzed using the same ordinal logistic or linear regression modeling techniques as described for primary and secondary endpoints, depending on the nature of the outcome. In addition, for binary outcomes, logistic regression modeling will be performed. Multiple imputation as implemented in the MICE (Multiple Imputation by Chained Equations) R package will be used for missing data. All variables included in the analyses of the primary and secondary endpoints will be included in the imputation models: 3‐month GOS‐E, hemorrhagic progression of the contusion, disability rating score (DRS) at discharge, 3‐month DRS, treatment group, TBI status, site, and numerous other variables outlined in the protocol. Ten imputed datasets will be generated, and results will be pooled according to Rubin's rules for both primary and secondary outcomes. To account for the possibility that missingness is related to the outcome, we will perform numerous sensitivity analyses to determine the robustness of our findings to non‐random missingness, outlined in detail in the trial protocol.

To guard against the possibility of continuing the trial if there is no benefit to the treatment, we plan to conduct an interim futility analysis when follow‐up is complete for *n* = 150 participants. If the conditional power estimate falls below 20%, trial continuation will be reassessed for futility. Conditional power will be calculated under the observed outcome distribution for these *n* = 150, as well as the three assumed scenarios examined (Table S4). The potential stopping of the trial will be discussed with the Data Safety Monitoring Board (DSMB) and regulatory consultants. The study includes no pre‐specified rules for stopping the trial early for efficacy, and all interim analyses are limited to safety monitoring.

## 6 RECRUITMENT

In 1996, the FDA created the exception from informed consent (EFIC) pathway for trials conducted on incapacitated individuals with life‐threatening conditions in which the time‐window needed for treatment is too narrow to obtain consent from the patient's surrogate before treatment.^33^ This trial will utilize the EFIC pathway given the time‐sensitive nature of administering VPA in TBI patients. Given that (i) patients will have an altered mental status secondary to TBI and thus be unable to provide consent before study enrollment and (ii) legally authorized representatives (LARs) are often not immediately available at the injury scene, sufficient justification exists for an exception from informed consent to administer (and study) an intervention that may have significant outcome benefits to this patient population. A past TBI trial (RAMPART) employing the EFIC pathway reported that the vast majority (83%) of subjects had positive views of enrollment, and were glad that they or their family members were enrolled in the EFIC trial; this trial will serve as a template for our study.^34^

At each participating center, a dedicated study team member will be available to obtain informed consent from patients or their LARs prior to enrollment, when it is feasible. If a patient is unable to give consent, no LAR is available, there is no evidence the patient has opted out of participation as a result of community consultation, and the patient meets all requisite eligibility criteria, they will be enrolled in our study. In this circumstance, a designated member of the research team will make frequent attempts as soon as feasible to contact a LAR or family member to provide information about the study and allow them the opportunity to make decisions regarding the patient's continued participation. In addition to consent protocols for patient enrollment, consent will be requested for follow‐up after hospital discharge to coordinate 3‐ and 6‐month post‐infusion follow‐up visits.

At each participating site, the pharmacy will prepare single‐unit dose bags for IV administration, which will be labeled with blinded study information including study number, subject number, and randomization assignment by a member of the dedicated study team at the participating site. The bags will be visually indistinguishable from each other, and the study team members will be blinded to the group allocation. Each participating site will employ the centralized, online, REDCap Electronic Data Capture System to determine the allocation of newly enrolled patients and label placebo or study drug IV preparations with blinded study information accordingly. Each study participant will be assigned a unique identification code that is de‐identified and specific to the site they were treated at, e.g. 01‐001 for patient one at site one, 02‐001 for patient one at site two, etc. The key to participant identification codes will be available to the study team and principal investigators and safeguarded as outlined in the study protocol. All of the trial data will be housed on central servers, protected and encrypted per Northwestern University policy and the study protocol.

Given that blinding occurs at the level of drug preparation by the participating site's dedicated study team, all trial participants, investigators, care providers, outcome assessors, and data analysts will be blinded. Available identifiers will include blinded study information, including study number, unique subject number, and randomization assignment. Investigators will have access to a sealed electronic envelope for enrolled patients under their care, to be opened in the event that knowledge of the administered substance becomes necessary for adequate patient care. Should the electronic envelope be accessed and unblinding occur, the date of opening and reason for opening will be recorded in the file.

## 7 DATA COLLECTION AND MANAGEMENT

Clinical study data obtained from screening, pre‐dose, dose administration, dose monitoring, and post‐treatment procedures will be entered and uploaded to Northwestern University's REDCap Electronic Data Capture System. REDCap is hosted and maintained at Northwestern University by the Northwestern University Clinical and Translational Sciences Institute (NUCATS). The Northwestern University Data Analysis and Coordinating Center will utilize REDCap to provide centralized data management, storage, and analytical support for the study, as well as serve as this trial's data coordinating center. All imaging and behavioral data will be uploaded to the Northwestern University Research Image Processing System (NURIPS), a secure environment designed for de‐identified storage, analysis, and management of medical imaging and associated data. NURIPS is supported by Northwestern University IT as well as the Feinberg School of Medicine IT and adheres to Northwestern University's latest policies and procedures for the storage and transit of data among approved users and groups. All data will be backed up and have restore points that go back 30 days.

This trial will be reviewed by a single Institutional Review Board (s‐IRB) housed at Vanderbilt University Medical Center, which is also one of the nine patient enrollment sites. All pertinent data for this trial will be gathered prospectively and entered into the REDCap Electronic Data Capture system, a data‐storage network frequently utilized by academic institutions participating in multi‐site trials. At Northwestern University, the Northwestern University Clinical and Translational Sciences Institute (NUCATS) hosts and maintains REDCap. Northwestern University Data Analysis and Coordinating Center (NUDACC) will provide the data management, storage, and analytical support necessary for the study. All data pertaining to this trial will be subject to weekly quality assurance review by a designated team to ensure that data is being handled in accordance with all requisite protocols and policies.

Clinical laboratory samples will be analyzed at the clinical sites from which they are collected in line with their respective hospital procedures. In addition, a total of 8 blood samples (20 mL each) will be collected serially at hours 1, 4, 6, 12, and 20–24 h from the time of infusion. Study personnel at each site will use 5 mL from each sample to perform coagulation studies at their institution and separate the rest of the blood into aliquots of serum, plasma, and peripheral blood mononuclear cells to be shipped securely in cold storage for future analysis at Northwestern University's bioanalytical laboratory. Blood samples for PK data will be collected and spun down, and plasma will be isolated at −70°C before being shipped to Northwestern University's bioanalytical laboratory on dry ice. Similarly, peripheral blood samples will be isolated for peripheral blood mononuclear cells (PBMCs) and plasma before long‐term cryogenic storage and shipped back to Northwestern University.

Given TBI studies' propensity for selective attrition of subjects who (i) are socioeconomically disadvantaged, (ii) have a history of substance abuse, and (iii) have violent injury etiologies, trials using the EFIC pathway must ensure fair and equitable demographic representation.^35^ We will address this existing medical disparity by ensuring that we include a diverse array of medical centers across our multi‐site study; we have selected sites that are geographically distinct and whose patient populations are diverse from one another socioeconomically, racially, and ethnically. Further, consent will be requested from patients to coordinate follow‐up visits, which will be conducted in person or using telehealth at approximately 3 and 6 months post‐infusion. By carefully observing enrollment patterns, adherence to the enrollment protocol, and completeness of follow‐up evaluation at all sites, we plan to minimize biases in this trial, particularly as it relates to retention. Should site‐level monitoring identify bias or discrepancies in follow‐up, we will implement prospective, corrective, and bias‐specific action such as but not limited to targeted retraining of research staff, implementation of enhanced retention strategies such as hiring dedicated follow‐up coordinators or increased follow‐up contact attempts, adaptive recruit adjustments, or temporary enrollment pause as a last resort. Due to the potential for survivorship bias, we will perform sensitivity analyses to examine the impact of early deaths on functional outcomes. This bias is further mitigated by the fact that this trial uses an intention‐to‐treat framework, and early deaths will be included in the primary and relevant secondary endpoints.

## 8 OVERSIGHT AND MONITORING

The establishment of an independent DSMB will take place for this trial. We have employed a regulatory consultant to aid us in the formation of the DSMB, including the identification and recruitment of its members, recording its activities, and delineating required reporting. The DSMB will include a chair, a clinical pharmacist, an ethicist, a trauma surgeon with expertise in TBI, an expert in patient safety, and a patient advocate. We will formalize protocols for DSMB activities, including a formal charter, writing meeting minutes, generating and distributing reports, and ensuring statistician support with the support of the consultant over the next 2 months.

With regards to the withdrawal of participants, stopping rules, and adverse events, safety will be monitored by a designated Safety Review Committee (SRC), which will consist of the principal investigator, co‐investigators, and medical monitor. The DSMB will review recommendations from the SRC monthly, to determine if any changes in safety monitoring procedures are necessitated or if the study must be stopped.

NUDACC under the leadership of Dr. Denise Scholtens will act as the data coordinating center to provide the data management, storage, and analytical support. NUDACC (https://www.feinberg.northwestern.edu/sites/nudacc/index.html) has the expertise to provide the full spectrum of services and support that is needed for high‐quality translational and clinical research. NUDACC supports the investigators, but administratively Dr. Scholtens reports to the Associate Vice President of Research/Senior Associate Dean for Clinical and Translational Research. All study data will be subjected to weekly quality assurance reviews including range, logic, and consistency checks. Data queries will be administered and monitored for resolution in collaboration with the study project manager. Experienced biostatisticians at NUDACC will analyze the data to ensure quality and completeness and generate the required reports. In addition, Broom Street Associates will provide regulatory and monitoring expertise for this project. The timely evaluation of ongoing and cumulative data allows early identification of site and study‐specific problem areas that warrant immediate remediation. Study monitoring reports will include data such as: actual versus expected study enrollment; demographic characteristics of participants by protocol‐driven parameters; enrollment status of participants (active, discontinued, loss to follow‐up, etc.); adverse events (AEs) by severity; details about participants experiencing SAEs, including SAEs resulting in death; clinical and laboratory results; and more. The NUDACC team will provide confidential reports for the DSMB, and separate (blinded) reports for the investigators. DSMB will provide independent oversight as described above.

All adverse events (AEs) occurring after participants have signed the informed consent or been enrolled in the study under the criteria will be recorded in subject case report forms. Each event will be described in detail along with start and stop dates, severity, expectedness, relationship to the investigational product, action taken, and outcome. Additionally, changes in the severity or seriousness of an event will warrant a new entry in a participant's case report form. Patients experiencing AEs will continue to be followed until the event has resolved or has been stable for 2 weeks and is not expected to worsen, at the discretion of the investigator. Reports of the same type of AE that are related to the study intervention that occur in four individuals will prompt a potential pause of enrollment in the trial for a safety review.

Prior to a participating site's activation, each site will submit a quality assurance plan delineating staff assigned to the protocol, roles, dedicated levels of effort, and participation in the implementation of the plan. Each site's quality assurance plan will serve as the site's plan for monitoring recruitment, eligibility criteria verification, protocol compliance, and AE management, among other metrics outlined in full in the trial protocol. The site pharmacy will be evaluated and monitored for correct storage, labeling, deployment, and recovery of patient‐specific investigational products. We will establish performance parameters including but not limited to timeliness and accuracy of data entry, timeliness of data reconciliation, and visit compliance, to track each site's ability to comply with quality standards. Performance benchmarks will be set for regulatory compliance, and the designated Project Manager will regularly review site performance reports and communicate with sites that fail to meet quality standards for the defined key performance indices. In addition, on‐site monitoring visits will be performed as needed, where a site's facilities and staff will be evaluated for the adequacy of quality standards.

This trial will implement the expertise of seasoned rehabilitation physicians to train the principal investigators (PIs) at each site, and to test their readiness prior to enrollment. The PIs, in turn, will train and certify their study team members. Regular quality checks will be performed during the course of the study to ensure that data gathering is being performed by trained and certified team members. During the first 3 months—and with the addition of each new study team member—the PI will review the recording of encounters and independently score the patient to ensure good interrater reliability.

Reports of site performance will be reviewed monthly by a centralized clinical operations team, which will directly communicate performance status with site PIs. Sites that fail to meet quality standards will be asked to develop remediation plans and be provided with improvement strategies based on the success of other sites. Sites that continue to fail to meet adequacy standards after 2 months will be placed on probation, resulting in more frequent on‐site monitoring visits, new enrollees being placed on hold, and/or being informed that their continued participation in this trial is no longer possible.

Any modification to the study protocol will not be implemented without agreement by both the sponsor and site investigator. Investigators will be able to implement a deviation from the protocol to eliminate immediate hazards to trial participants without prior IRB approval if deemed necessary. In this event, the implemented deviation from the protocol and the reasons for it will be submitted to the IRB or sponsor as soon as possible. Additionally, any amendments or addenda to the protocol must be approved by the common Institutional Review Board (c‐IRB) prior to implementing changes in the study. The PI will be responsible for keeping the c‐IRB and the FDA apprised of the study's progress and any changes made to the protocol at least once per year.

Both positive and negative results from this trial will be published in international peer‐reviewed journals as soon as possible. Additionally, trial results will be made available publicly on a dedicated study site hosted by the University of Alabama at Birmingham. The datasets used and/or analyzed during the current study will be made available from the corresponding author upon reasonable request.

## 9 CONCLUSIONS

TBI is a prevalent and serious injury that affects people of all ages and has important implications within military medicine. The identification of a pharmacological agent that is readily available, easy to transport, shelf‐stable, and can attenuate the secondary injury processes following TBI would have drastic implications in both civilian and military systems. If successful, VPA could become the first pharmacotherapy for TBI, offering a cost‐effective, easily administered treatment option in a medically austere environment.

## AUTHOR CONTRIBUTIONS

MV: Manuscript design and development. ML: Design, review, and editing of the manuscript. SL: Protocol development, trial coordination, review, and editing of the manuscript. UB and BW: Reviewing and editing of the manuscript. ZD: Reviewing and editing of the manuscript. AA: Reviewing and editing of the manuscript. NG: Development and optimization of statistical methods and protocols. DS: Development and optimization of statistical methods and protocols. HA: Principal investigator, and critical review of the manuscript. All authors have read and approved the final manuscript.

## FUNDING INFORMATION

The projects reviewed here were supported by multiple sources. Dr. Alam acknowledges research support provided by numerous grants from the Office of Naval Research (including N000140910378 and N000141310071), the US Army Medical Research and Materiel Command (W81XWH‐09‐1‐0520; W81XWH‐17‐C‐0246; and W81XWH‐17‐1‐0701), the Defense Advanced Research Projects Agency (W911NF‐06‐1‐0220), and the National Institutes of Health (R01GM084127). The Multi‐Institutional Phase 2/3 Trial of Valproic Acid in Patients with Moderate to Severe Traumatic Brain Injury is being supported by an award from the Congressionally Directed Medical Research Program (CDMRP) to Dr. Alam (Award no. HT94252410241). The opinions and assertions contained herein are the private ones of the authors and are not to be construed as official or reflecting the views of the Department of Defense at large.

## CONFLICT OF INTEREST STATEMENT

The authors declare that they have no competing interests.

## ETHICS STATEMENT

The full trial protocol, two‐part informed consent form, relevant supporting information, and all types of patient recruitment or advertisement information will be submitted to the cIRB for review and approval prior to initiation. Approval was granted by the FDA on 01/06/2025 (IND # 146723). This manuscript does not contain individual personal data from patients. The authors declare that they have no competing interests. This manuscript was drafted using the SPIRIT reporting guidelines.^36^

## Supporting information


**TABLE S1:** Known drug interactions with valproic acid (VPA).
**TABLE S2:** SPIRIT 2013 Figure for the VIBRANT trial. Abbreviations: DRS, disability rating score; GCS, Glasgow coma scale; GOS‐E, Extended Glasgow outcome score; HPC, Hemorrhagic progression of the contusion; ICP, intracranial pressure; PK/PD, pharmacokinetics/pharmacodynamics.
**TABLE S3:** Power calculation details across three scenarios (A, B, C).
**TABLE S4:** GOS‐E distributions across three scenarios (A, B, C).

## Data Availability

The data that support the findings of this study are available on request from the corresponding author. The data are not publicly available due to privacy or ethical restrictions.
