# Supplementary material for: Valproic acid for treatment of traumatic brain injury: Study protocol for the VIBRANT prospective randomized trial
Source: Transfusion. 2025 Dec 20;66(Suppl 1):S266–77. doi: 10.1111/trf.70029 (PMC13184415; doi:10.1111/trf.70029)
Supplement: Supplementary file 1 — TABLE S1: Known drug interactions with valproic acid (VPA). TABLE S2: SPIRIT 2013 Figure for the VIBRANT trial. Abbreviations: DRS, disability rating score; GCS, Glasgow coma scale; GOS‐E, Extended Glasgow outcome score; HPC, Hemorrhagic progression of the contusion; ICP, intracranial pressure; PK/PD, pharmacokinetics/pharmacodynamics. TABLE S3: Power calculation details across three scenarios (A, B, C). TABLE S4: GOS‐E distributions across three scenarios (A, B, C). [file TRF-66-S266-s001.docx]

Supplemental Information for:

**Valproic Acid for Treatment of Traumatic Brain Injury: Study Protocol for the VIBRANT Prospective Randomized Trial**

Maxime A. Visa^1^; Marjorie R. Liggett^1^; Sharnia Lashley^1^; Umar Bhatti^1,2^; Zaiba A. Dawood^1^; Alvin Anand^1^; Nathan P. Gill^3^; Denise M. Scholtens^3^; Bowen Wang^1^, Hasan B. Alam^1^

^1^ Department of Surgery, Northwestern University Feinberg School of Medicine, Chicago, IL

^2^ Department of Surgery, Cedars Sinai, Los Angeles, CA

^3^ Department of Preventive Medicine, Feinberg School of Medicine, Northwestern University, Chicago, IL

**Table S1:** Known drug interactions with valproic acid (VPA).

| **Timepoint** | Enrolment (t0) | Allocation (≤120 min post-injury) | Post-Allocation (24-168 hours) | Discharge | 3 months post-injury | 6 months post-injury | Enrolment Completion |
| --- | --- | --- | --- | --- | --- | --- | --- |
| **ENROLMENT:** |  |  |  |  |  |  |  |
| Eligibility screen | X |  |  |  |  |  |  |
| *Informed consent | X |  |  |  |  |  |  |
| Randomization |  | X |  |  |  |  |  |
| **INTERVENTIONS:** |  |  |  |  |  |  |  |
| Control: Standard of care + Normal Saline |  | X |  |  |  |  |  |
| Experimental: Standard of care + Low-dose VPA (50 mg/kg) |  | X |  |  |  |  |  |
| Experimental: Standard of care + High-dose VPA (100 mg/kg) |  | X |  |  |  |  |  |
| **ASSESSMENTS:** |  |  |  |  |  |  |  |
| GCS | X |  | X |  |  |  |  |
| GOS-E |  |  |  |  | X | X |  |
| HPC |  |  | X |  |  |  |  |
| DRS |  |  |  | X | X | X |  |
| PK/PD Data |  |  | X |  |  |  |  |
| ICP |  |  | X |  |  |  |  |
| Statistical Analysis |  |  |  |  |  |  | X |

**Table S2:** SPIRIT 2013 Figure for the VIBRANT trial. Abbreviations: GCS: Glasgow coma scale, GOS-E: Extended glasgow outcome score, HPC: Hemorrhagic progression of the contusion, DRS: Disability rating score, PK/PD: Pharacokinetics/Pharmacodynamics, ICP: Intracranial Pressure.

*This trial is being executed under an approved exception from informed consent (EFIC). Pre-randomization informed consent may be unlikely.

| **Parameters** | **Scenario A** | **Scenario B** | **Scenario C** |
| --- | --- | --- | --- |
| Level of Significance (α) | 0.05 | 0.05 | 0.05 |
| Sample Size | 432 | 432 | 432 |
| No. treatment groups | 3 | 3 | 3 |
| Median GOS-E in Control Group | 5.0 | 5.0 | 5.0 |
| Median GOS-E in Treatment Group | 5.7 | 5.8 | 5.9 |
| Attrition | 5% | 5% | 5% |
|  |  |  |  |
| **Power (1-β)** | 84% | 80% | 85% |

**Table S3**: Power calculation details across three scenarios (A, B, C).

| **GOS-E** | **Control** | **Scenario A** | | **Scenario B** | | **Scenario C** | |
| --- | --- | --- | --- | --- | --- | --- | --- |
|  |  | *50 mg* | *100 mg* | *50 mg* | *100 mg* | *50 mg* | *100 mg* |
| **1** | .09 | .07 | .05 | .08 | .07 | .07 | .06 |
| **2** | .09 | .07 | .05 | .07 | .06 | .07 | .06 |
| **3** | .15 | .09 | .04 | .10 | .06 | .10 | .06 |
| **4** | .09 | .06 | .04 | .06 | .05 | .07 | .05 |
| **5** | .07 | .10 | .13 | .10 | .10 | .10 | .12 |
| **6** | .21 | .23 | .25 | .20 | .20 | .18 | .16 |
| **7** | .18 | .22 | .25 | .23 | .27 | .22 | .24 |
| **8** | .12 | .16 | .18 | .17 | .20 | .20 | .25 |

**Table S4:** GOS-E distributions across three scenarios (A, B, C).
